# Zinc Slag as a Partial or Total Replacement for Mineral Filler in Warm Mix Asphalt and Its Effects on Self-Healing Capacity and Performance Characteristics

**DOI:** 10.3390/ma15030736

**Published:** 2022-01-19

**Authors:** Mansour Fakhri, Sajad Javadi, Alireza Sassani, Mohsen Torabi-Dizaji

**Affiliations:** 1 Department of Civil Engineering, K. N. Toosi University of Technology, Tehran 1996715433, Iran; sajadjavadi2012@gmail.com; 2 Institute for Transportation, Iowa State University, Ames, IA 50010, USA; asassani@iastate.edu (A.S.); mohsent@iastate.edu (M.T.-D.)

**Keywords:** warm mix asphalt, zinc slag filler, self-healing, moisture susceptibility, rutting

## Abstract

Utilizing the self-healing properties of asphalt materials is a way to improve the service life of asphalt pavements. Enhanced self-healing capabilities can be achieved through mixture modification. Using waste or by-product materials to modify asphalt mixtures can provide further environmental benefits. However, with a given mixture modification method, the resulting materials should be adequately vetted to ensure that enhanced self-healing capability is not attained at the expense of the mixture’s overall performance. This research aims to investigate the feasibility of using zinc slag filler to enhance the self-healing properties of warm mix asphalt (WMA) and evaluates the rutting susceptibility and moisture-resistance of the slag-modified mixtures. To this end, zinc slag filler was used to replace a portion of the mineral filler at different replacement rates in WMA mixtures. Self-healing capabilities of the resulting mixtures were studied under microwave induction heating. The influence of zinc slag modification on asphalt mixture’s characteristics and conventional performance indicators were evaluated by the Texas boiling test, the three-point bending test, and the Kim test (a deformation test). Also, the adhesion between bitumen and aggregate was evaluated using the broken sample from the three-point bending test and digital image analysis. The results of self-healing tests demonstrated that the heat generation capability of the specimens increased with filler replacement rate, such that the specimens with 100% of the mineral filler replaced with slag showed the highest heating performance. Zinc slag filler showed the potential to improve the moisture resistance of WMA by enhancing aggregate–bitumen adhesion and thus reducing stripping. The slag-modified WMA samples exhibited better tensile strength ratio (TSR) and deformation resistance than their non-modified equivalents.

## 1. Introduction

Utilizing waste or by-product materials in asphalt pavements for functional, environmental, or economic benefits has been long studied and encouraged. Some recycled materials such as recycled asphalt pavement (RAP) and recycled asphalt shingle (RAS) are so commonly used in asphalt mixtures that have gained a standard asphalt mixture component status that their use is not being questioned [1]. Various types of relatively unconventional or less common waste, by-product, or otherwise recycled materials such as waste tire thread mesh, recycled waste lime, municipal solid waste incineration ash, waste foundry sand, recycled carbon fiber, and different metallic waste/by-product metallic materials have been used to modify asphalt-based composites for different purposes [2,3,4,5,6,7,8,9]. Modified asphalt materials are primarily made to achieve improved performance characteristics; however, asphalt-modifying agents can also impart to the composite new properties. Waste materials for asphalt modification can be derived from very diverse base materials sourced from different industries or origins; this gives rise to all sorts of unknowns and challenges in the application of the end products that, in this case, are modified asphalt composites. Adding a given material to asphalt mixture may be meant to enhance or instill certain properties but can affect—positively or negatively—other aspects of the asphalt composite’s or pavement’s behavior [9].

Metallic waste additives in the asphalt have been studied primarily with a focus on the thermal and electrical performance of the products [3,4,6,9]. Notably, Norambuena-Contreras et al. [10] investigated the effects of waste metal additives such as metal fibers and steel shaving on the thermal properties and electrical conductivity of asphalt mixtures. The study also performed a thermal healing test with microwave stimulation and reported that waste metal additives improved crack healing and strength recovery. Another study on the self-healing properties of asphalt by microwave heating [11], used RAP and metal fibers. Using RAP in the mixture increased the stiffness modulus and decreased self-healing, while the addition of metal fibers compensated for the RAP’s effect by improving induction heating capability. The study reported an increase of porosity as the drawback of using steel fibers but concluded that the mixtures showed desirable healing performance under microwave induction heating.

Zinc-bearing industrial waste can release zinc and its significantly more toxic oxidation product (ZnO) into the environment, posing environmental and health concerns, especially if they are deposited for a long time and allowed to oxidize [12]. Recycling or reusing zinc-bearing waste helps reduce the environmental concern associated with them by making good use of potentially toxic waste. Using zinc products in the form of nanoparticles, slag, or chemical compounds to modify bituminous materials has precedent in the existing literature [13,14,15,16,17,18]. Ouyang et al. [16] reported that modifying asphalt with zinc dialkyl-dithiophosphate (ZDDP) resulted in improved aging resistance and oxidation resistance. The aging resistance improvement was attributed to the ZDDP suppressing carbonyl formation and thereby enabling asphalt molecules to maintain unchanged molecular weight over a longer time. Also, ZDDP acted as an oxidation retarding asphalt oxidation by radical scavenging and inhibition of peroxides. Studies reported significant improvement of moisture resistance by modifying asphalt with zinc nanoparticles [17,18]. Hamedi et al. [17] used zinc oxide nanoparticles as an anti-stripping agent in hot mix asphalt (HMA) and reported a significant reduction of moisture damage under freeze–thaw cycles. Saltan et al. [18] also confirmed the desirable effect of ZnO nanoparticles (ZnONP) on moisture resistance of HMA, while reporting degradation of fatigue performance by ZnONP modification. However, contrary to the ZnONP, zinc slag may reduce moisture resistance according to Aktas and Aslan’s study [19] that studied the effects of substituting the regular aggregate filler with zinc slag filler in Stone Mastic Asphalt (SMA). The study, however, showed an improvement of Marshal resistance of the SMA mixture by slag replacement.

Warm mix asphalt (WMA) has gained popularity in recent years because of its environmental and practical benefits, and technologies for producing and implementing WMA have evolved rapidly [20,21]. Using WMA enables reducing asphalt temperature by 20–30 °C, while most important performance parameters such as rutting resistance remain comparable to HMA [22] or have even improved. The reduction of asphalt temperature can result in a reduction of air pollution by up to 24%, fossil fuel consumption by up to 18%, and chemical vapors by 10%. Consequently, replacing HMA with WMA reduces the carbon footprint of asphalt pavements by 15% [20]. WMA has a higher moisture resistance than an equivalent HMA, and studies have shown that its moisture resistance can be improved by incorporating anti-stripping agents or appropriate metallic additives [23]. For instance, Fakhri and Ahmadi [21] and Ziari et al. [24] showed that electrically conductive metallic slags could enhance the moisture resistance of WMA.

In Liu et al. [22], the effects of metal fibers on the thermal characteristics—including thermal conductivity, thermal diffusivity, and specific heat—of HMA and WMA were investigated, and their healing performance under microwave induction heating was evaluated. The study revealed that WMA could exhibit better rutting performance but slightly lower low-temperature cracking than HMA. WMA showed slightly lower strength recovery than HMA in the self-healing; this behavior was attributed to the presence of residual active ingredients in WMA, reducing the optimal induction heating temperature and undermining strength recovery. Also, Liu et al. speculated that the formation of foams at the crack surfaces could prevent crack closure in WMA. Safe application of any material in asphalt production calls for adequate testing and vetting to ensure that the final product’s performance characteristics meet the expectations. Performance testing has been an integral part of the asphalt mixture design process since the 1920s. Hubbard-Field method was the earliest documented method for performance testing, followed by the Marshall method and Hveem method in the 1930s. Later in the 1980s and 1990s, the Superpave method incorporated the performance testing concepts developed under the previous methods and the determination of optimum asphalt content into the asphalt mixture design process. The performance testing component of Superpave is often overshadowed by its volumetric component because it has been deemed complex, time-consuming, or costly. However, the performance tests developed or promoted by Seperpave are widely utilized for performance evaluation of asphalt mixtures, especially after the development of performance-related specifications (PRSs) during the 2000s [25]. Asphalt pavement performance strongly correlates to the volumetric design of asphalt mixture, the properties of mixture components (i.e., bituminous binder, aggregates, and fillers), and the in-situ density. The bituminous binder grade, asphalt binder content, type and gradation of aggregate, type and amount of fillers, and the mixture’s void ratio can significantly influence the mixture’s performance. These characteristics can be experimentally evaluated through rutting, fatigue cracking, low-temperature cracking, moisture sensitivity, and durability tests. The density—and void ratio—of asphalt pavement with a certain mix design is governed by the quality of compaction. Laboratory compaction methods, including vibration, kneading, impact, and gyration, attempt to simulate field compaction. In the Marshall mix design procedure (adopted in this research), the suitable compaction effort to best simulate field compaction is represented by a certain number of blows that is determined during the mix design process. Its Superpave equivalent is the number of gyrations [26,27,28,29].

The performance concepts involved in the standardized performance tests can be utilized to establish various performance indicators/indices used to project field performance from laboratory results. For instance, Javilla et al. [27] used the wheel tracking test results to evaluate different rutting indicators of asphalt mixtures. Another notable example is found in Polaczyk et al. [28], where the influence of aggregate interlocking on asphalt performance was investigated through rutting resistance, represented by Flow Number, and fatigue cracking, represented by the IDEAL CT test result. The research reiterated the direct relation between compaction and performance and suggested that the optimum gyratory compaction to achieve aggregate interlocking point is obtained from the Flow Number and Cracking Tolerance Index. Low-temperature cracking and moisture resistance are other performance indicators commonly used to evaluate asphalt mixtures, especially non-conventional mixtures containing a modified binder, specially graded aggregates, differently sourced aggregates, or additives. Moisture damage manifests as the failure of aggregate–binder adhesion (stripping) or binder cohesion. Moisture damage is critical in warm mix asphalt (WMA) due to the aggregate conditions and the presence of additives [30]. Additive materials, whether used as a reinforcing component, binder modifier, or filler, have a high potential to influence the bond between the aggregate and bituminous binder. Hence, studying the characteristics of modified asphalt mixtures calls for careful examination of moisture resistance, especially when WMA is involved [31,32,33].

Bituminous materials are known for possessing heat-activated self-healing properties, the level of which depends on the material’s physical and chemical characteristics. Various mechanisms have been provided for the healing process in bituminous materials, mostly based on Van Der Waals forces and intermolecular hydrogenic interactions. The Van Der Waals forces are the driving force behind molecular interdigitation, enabling partial or complete closure of the existing cracks under heat treatment. The intermolecular hydrogenic interactions govern the crack’s surface energy that is a significant factor in strength recovery [34]. According to Kringos et al.’s chemomechanical model [35], the development of cracks in bituminous materials is primarily caused by phase separation and the increase of surface energy at the interface of different phases. On the other hand, the separated phases can re-homogenize by an increase of energy level—possibly—through temperature rise or mechanical force. Reassuming the phase uniformity helps the bituminous material recover from cracks and regain all or a portion of its mechanical characteristics. However, asphalt material can marginally self-heal at ambient temperatures, requiring heat treatment at elevated temperatures to complete the recovery process.

Research has shown that temperature is the primary factor in the self-healing of bituminous materials [36,37]. Consequently, the quality of heat transfer through asphalt is a significant factor in the healing ability of asphalt materials. The other critical factors influencing the self-healing capability of bituminous include the rheological properties and the fractional chemical compositions of bitumen. As a result of aging, the viscosity of bitumen increases, and its self-healing capability is decreased. The extent of the influence of aging on asphalt binder’s healing capacity varies by material and aging conditions [38,39]. Previous studies have investigated the heat treatment of asphalt pavements utilizing the self-healing properties of the bituminous binder and extending the pavement’s service life. Heat treatment of asphalt mixtures containing mineral aggregates triggers microcrack closure in the bituminous binder while also healing macrocracks through the improved rheology of the viscoelastic binder. Asphalt binder being a viscoelastic material, exhibits decreased viscosity at elevated temperatures flowing through macrocracks and contributing to the asphalt pavement’s healing [40,41,42].

Heat generation through electric current-based or microwave induction methods has been long considered for use in asphalt pavements and is intensively studied. These methods have shown effectiveness in healing and deicing applications if matched with the appropriate materials [43,44]. The current-based methods include resistive (Joule) heating and inductive (Eddie current) heating. Microwave induction heating can be applied with conventional asphalt, while the current-based methods require electrical conductivity levels above those seen in conventional asphalt materials. However, the efficiency of both methods is directly related to the material’s electrical and heat conductivity. Higher electrical conductivity results in stronger heat generation using any of the methods, and a higher heat conductivity improves the effectiveness of the practice by enabling faster heat transfer through the bulk of the material and enhancing the heat distribution uniformity.

Resistive heating has practical advantages, especially in pavement deicing applications, because it is easier to implement and has been intensively studied before. Nevertheless, microwave induction heating provides more uniform heating that is advantageous in healing applications. Heat generation in the current-based heating follows the current path through the material and does not involve volumetric heating. The heterogeneity of asphalt pavement means that the electrical conductivity is highly variable through the material’s volume. Hence, the electric current flows through the material in separate paths with varying current magnitudes leading to non-uniform heat generation. Note that, despite being considerably more uniform than current-based heating, microwave induction heating is also susceptible to variabilities due to the possible irregularities in the electromagnetic field and the heterogeneity of material characteristics such as dielectric properties, polarity, material state, atomic/molecular weights, and density [44,45,46,47]

As mentioned above, while microwave induction heating does not necessarily require electrically or thermally conductive asphalt, higher electrical or thermal conductivity results in better induction heating performance. Early studies on the microwave induction heating of asphalt materials in the late 1980s and early 1990s showed that the method would have undesirable efficiency with conventional asphalt materials. The microwave absorption at or near the surface of non-conductive asphalt specimens ranged between 4% and 5% and that is too low for either deicing or healing applications [48]. Therefore, later studies were steered towards considering electrically conductive asphalt (bituminous composites) to improve the efficiency of microwave induction heating [49]. Electrically conductive asphalt mixtures are obtained by mixing electrically conductive additives with conventional or otherwise modified asphalt mixtures. The conductive additives are typically metallic or carbon-based materials in the form of granular particles or fibers, and they can impart electrical conductivity to the asphalt while also improving its thermal conductivity

The existing literature has identified zinc products such as zinc slag and ZnONP as candidate additives for the enhancement of WMA. The electrical conductivity of zinc products makes them particularly desirable for induction-heating applications; incorporating zinc in asphalt materials imparts electrical conductivity to the mixture, increasing microwave absorption and improving the induction heating capability. Zinc slag has an environmental and economic advantage over other zinc products because it is an industrial waste with potential long-term hazardous nature if released into the environment. The overarching objective of this research is to investigate the feasibility of using zinc slag as a filler in WMA to improve microwave-induced healing and assess its influence on asphalt performance. Asphalt performance indicators studied under this research included rutting resistance, moisture resistance evaluated for WMA mixtures containing zinc slag in different fractions. Healing performance was evaluated through strength recovery of asphalt samples under microwave-induction heating. It is worth mentioning that some other aspects of the performance of this type of asphalt mixtures, including fatigue and low-temperature cracking, durability, and life-cycle cost analysis, are in progress under a different research plan, and the authors expect to publish the results in the future.

## 2. Materials and Methods

### 2.1. Materials

WMA samples were prepared with virgin bitumen with a penetration grade of 60/70, with characteristics as shown in Table 1. The mix designs according to the Marshall method are shown in Table 2.

**Figure 1:**
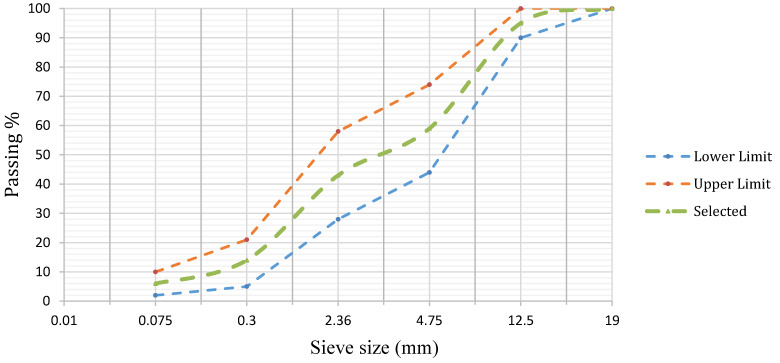
The Gradation of Aggregates in the WMA sample.

Aggregates were crushed limestone with physical properties and gradation, as shown in Table 3 and Figure 1, respectively. Zinc slag filler passing #200 sieve was used to replace a portion of the mineral filler (dolomite limestone) at three different replacement fractions of 35%, 70%, and 100% by volume. These slag contents were selected as in the work of Nabiun and Khabiri [50]. Chemical compositions of the mineral filler and the slag filler were determined using X-ray fluorescence (XRF) spectroscopy. The chemical analysis results are given in Table 4. As seen in this table, the zinc slag is rich in ZnO, and contains some Fe_2_O_3_, a chemical composition that promises electrical and thermal conductivity desirable for the purpose of this study.

### 2.2. Experimental

The experimental plan was developed, having in mind the climatic conditions with extreme temperature gradients between day and night. These environmental conditions were selected to represent the environmental conditions in Iran, where the research was conducted. Large differences between day and night temperatures, especially when associated with freeze–thaw cycles, is one of the principal factors contributing to asphalt pavement deterioration. A large temperature difference between summer (hot season) and winter (cold season) generally represent conditions in which asphalt pavements are susceptible to rutting during summer and to low-temperature cracking during winter. In this research, moisture susceptibility, rutting susceptibility, and freeze–thaw resistance of the slag-containing WMA samples were evaluated to investigate their performance in such climatic conditions. The healing capability was assessed to test the repair and rehabilitation potential by means of microwave induction heating.

WMA samples were prepared by adding 1.5% (by weight of bitumen) Fisher–Tropsch wax to the mixture. The Marshall samples were designed and prepared according to the ASTM-D 1559 standard practice, with a compaction of 75 blows per side of the sample to determine the optimum bitumen content for different percentages of zinc slag filler before the experiments. Production and compaction temperatures were 130 and 120 °C, respectively.

### 2.3. Moisture Resistance Tests

#### 2.3.1. The Texas Boiling Test

The Texas boiling test as described in ASTM D3625 (Standard Practice for Effect of Water on Bituminous-Coated Aggregate Using Boiling Water) was employed to evaluate the stripping potential of uncompacted samples. The samples were placed in boiling water for ten minutes and shaken by a glass rod for ten seconds every three minutes. The mixture was then removed from the water and placed on a white surface to detect the area coated by the bitumen [51]. High-resolution images were taken from the samples in which the spots with black color showed the area that breakage was within the bitumen, and spots with the color of aggregate indicated the areas that bitumen was detached from aggregates. The reduction of the aggregate surface coated by bitumen as a result of submersion in boiling water, calculated by Equation (1), is used as an indicator of moisture damage.

(1) $$X\%=\left[\frac{A\%-B\%}{A\%}\right]\times 100$$

where, X is the reduction of the surface area covered by bitumen, A is the total surface area of the sample, and B is the surface area not covered by bitumen. The method is based on visual analysis of the aggregate surface coated by bitumen using a color contrast. This analysis was performed in this study using ENVI (version 5.5.1., L3Harris ™ Geospatial, Herndon, VA, USA) and MATLAB (version R2020b, MathWorks, Natick, MA, USA) digital image processing tools.

#### 2.3.2. The Indirect Tensile Strength Test (AASHTO T283)

The modified Lottman indirect tensile method (AASHTO T283) investigates changes in indirect tensile strength while samples are waterlogged. Two series of compacted cylindrical samples with 6–8% mean air void were prepared. For the uniform-gradation mixtures, samples were placed in plastic bags, completely isolated, and placed in a water bath at 25 °C for 2 h. Then, the indirect tensile strength test was performed. The second set of specimens was subjected to a submerged vacuum to achieve a water absorption of 55–80% (by volume). Then, freeze–thaw cycles were applied to the specimens prior to the indirect tensile test, which was performed at 25 °C. In the indirect tensile test, the specimens were positioned supine between the loading jaws of the compression loading device, and the load was applied at a constant deformation rate of 50 mm/min until failure by breakage. The tensile strength of each specimen was calculated from the maximum load and specimen’s dimensions using Equation (2). The tensile strength ratio (TSR), was calculated as the ratio of wet and dry ITSs, as shown in Equation (3). A TSR of 80% or greater is considered to be satisfactory.

(2) $$\mathrm{ITS}=\frac{2P}{\pi \;\times \;D\;\times \;t}$$

ITS: Indirect Tensile Strength $\left[\mathrm{kPa}\right]$. 

P: Maximum load $\left[N\right]$. 

D: Sample diameter $\left[m\right]$. 

T: Sample thickness $\left[m\right]$.

(3) $$\mathrm{TSR}=\frac{{\mathrm{ITS}}_{\mathrm{wet}}}{{\mathrm{ITS}}_{\mathrm{dry}}}.\;$$

where ITS_dry_ is the mean tensile strength of the dry state and ITS_wet_ is the mean tensile strength of the samples under freeze–thaw cycles. The greater the value of this ratio, the greater the resistance to moisture damage.

The ITS test specimens were used after the test for yet another moisture resistance test. WMA is generally known to satisfy the required bond strength between the aggregate and bitumen; however, the bond strength may be low due to the low temperature of production [52]. In general, there are two types of moisture damage in asphalt pavements: those driven by a loss of adhesion between bitumen and aggregates and those caused by a failure within the bitumen matrix due to the softening of the mastic [53]. In this study, the former is investigated using the broken specimens from the ITS experiment after Fakhri et al. [54]. As mentioned before, the ITS test continued after mechanical failure until the sample was completely broken, as per AASHTO recommendation. Then, high-resolution digital images were taken from the separated crack surfaces and analyzed to evaluate the adhesion between the mastic and the aggregates. As described earlier, image processing was performed using ENVI and MATLAB.

**Figure 2:**
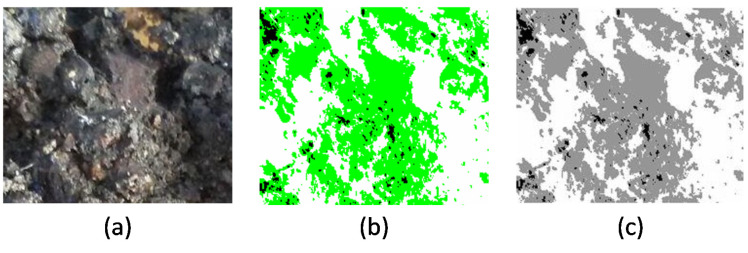
Image processing with ENVI and MATLAB (fractured samples subjected to indirect tensile testing): (**a**) image after test, (**b**) image processed by ENVI, and (**c**) image processed by MATLAB.

In the raw images of the fracture surfaces of the specimens, fractured aggregates were seen in white color, and the bitumen rupture zones were seen in and brown. Images were first segmented using ENVI, and then the output images were processed at grayscale by MATLAB, as shown in Figure 2. In the grayscale MATLAB images, gray zones represent adhesion failure, black zones show broken aggregates, and white zones are the intact bituminous matrix. The rate of sample failure was calculated as the surface fraction representing each failure type.

### 2.4. Deformation Test (Kim Test)

Given the importance of rutting damage in asphalt mixtures, especially in WMA, the ability to evaluate this type of failure with simple and rapid tests could be instrumental to evaluating the performance of asphalt mixtures. Deformation strength is one of the simplest tests for evaluating the rutting of asphalt mixtures; a static test is conducted to evaluate the rutting potential of asphalt, and the mixture with higher deformation strength is identified as displaying higher resistance to rutting [55,56]. According to Fakhri et al. [57], considering the strong correlation between static deformation strength and rutting resistance revealed by the Wheel Track test, the use of static deformation strength is recommended due to the simplicity of the test.

**Figure 3:**
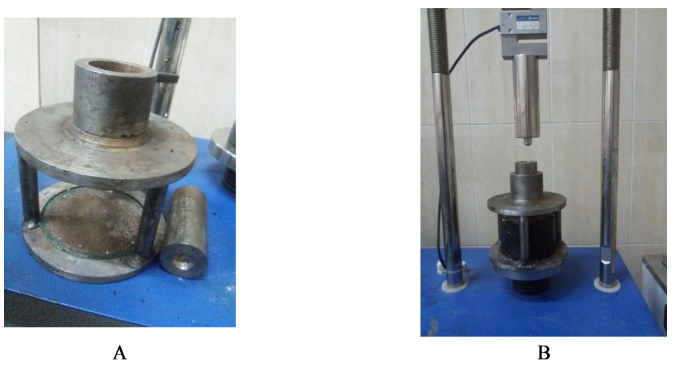
(**A**) Kim test mold and (**B**) typical failure modes.3.4. Self-healing Tests.

This test begins by placing the sample (using the Marshall method) at 60 °C for 30 min. After drying the surface, the sample is placed in the loading frame (Figure 3). The vertical static load is then applied at a pressure of 50.8 mm/min on the top plate. The loading jaw has a smooth bottom surface, a curved edge, a diameter of 40 mm, and a radius of curvature of 10 mm. The ultimate force is recorded against deformation, and deformation strength is calculated using Equation (4).

(4) $$S_{D}=\frac{0.32P}{\left[10\;p+\sqrt{20y-{\;y}^{2}}\;\right]}.\;$$

where S_D_ is the deformation strength $\left[\mathrm{MPa}\right]$, P is the maximum load $\left[N\right]$, and $\left[y\right]$ is vertical deformation $\left[\mathrm{mm}\right].$

### 2.5. Self-Healing Test

**Figure 4:**
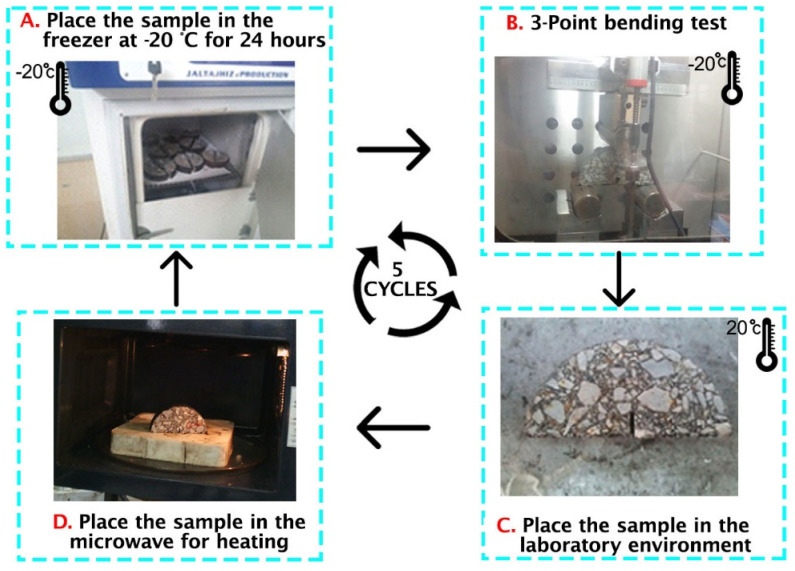
Steps involved in microwave self-healing.

The three-point bending test is employed to evaluate the self-healing properties of cracked asphalt mixtures containing metallic waste materials under microwave induction heating. Using semicircular specimens, the strength was measured through the Marshall method, in which the specimens were placed at −20 °C for 24 h prior to testing to capture brittle fracture. A notch with 3-mm width, and10-mm depth was cut at the center of the test specimen, as shown in Figure 4C. The notch is meant to guide the vertical loading path in the three-point bending test so that the crack is initiated at the notch tip. The process of measuring the rate of crack healing involves the following steps:

1. The semicircular specimens are mounted on two separate cylindrical rollers that are 80 mm apart. A monotonic (uniform) load of 0.5 mm/min is applied to the specimen at the semicircular arc’s midpoint. The loading machine is equipped with a 20 kN load cell.

2. Once the first bending test is performed, the broken asphalt specimens are removed from the machine chamber. To ensure that surface moisture is completely vaporized, the specimens are kept at room temperature for two hours until they reach a temperature of 20 °C. Microwave induction heating is then employed to trigger self-healing in the asphalt specimens. Microwave exposure durations were 40, 50, and 60 s following previous studies [58,59]; initially, durations of 70 and 30 s were considered but dismissed because most asphalt specimens deformed after 70 s of heating and 30 s was found to be inadequate for initiation of healing. All tests were performed in a microwave oven with 800 W power, 2.45 GHz frequency, and 120 mm wavelength.

3. Calculation of strength recovery was performed after Norambuena-Contreras et al. [10]. Following the completion of heating, the specimens were kept at room temperature for 2 h. They were then placed at −20 °C for 24 h, and the bending test was repeated to complete the healing cycle. According to Equation (5), the healing level for each semicircular specimen is obtained by dividing the maximum load after each step of the healing process (Fhealed) by the initial maximum load (i.e., the maximum load measured prior to the first healing cycle):

(5) $$HL=\frac{F_{\mathrm{healed}}}{F_{\mathrm{initial}}\;}$$

A total of 5 crack healing cycles were performed for each asphalt specimen in order to evaluate the efficiency of the healing process.

**Figure 5:**
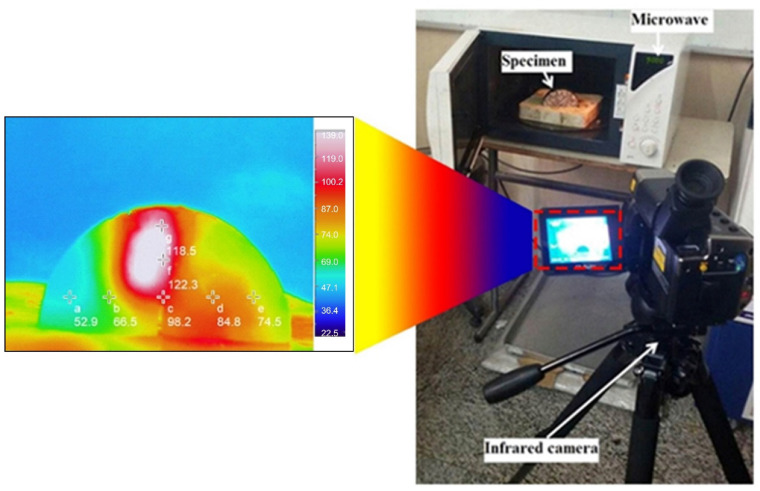
Thermal Imaging of the SCB Sample.

The surface temperature of semicircular specimens was measured after each heating period using a 640 × 480-color full-color infrared thermal imaging camera with a 30-Hz transfer rate. Thermal images were captured from the specimen’s surface in 10-s intervals, and the images were used to obtain the average surface temperature. Figure 5 shows the test laboratory setup used for assessing the heat generation efficiency of the specimens. Note that this study was a laboratory investigation, while real-world applications will require more powerful induction heating systems capable of heating the pavement surface at a larger scale. However, for laboratory test purposes, the system used in this study provides adequate power. Previous laboratory-scale studies have used the microwave at equal or lower powers than this study. For example, Fakhri et al. [60] used a microwave with the following characteristics: 800-W output power and frequency of 2.45 GHz for heating the asphalt mixtures containing steel shaving and tire steel fibers or Xu et al. [61] employed the microwave with 8300-W power and frequency of 123 kHz for induction heating.

## 3. Results

Marshall test results for each type of mixture are presented in Table 5. Considering Marshall Stability, the optimum rate for replacement of mineral filler with slag filler was 70%. The results showed that replacing 100% of the mineral filler with slag filler would lead to a reduction of Marshall resistance.

**Figure 6:**
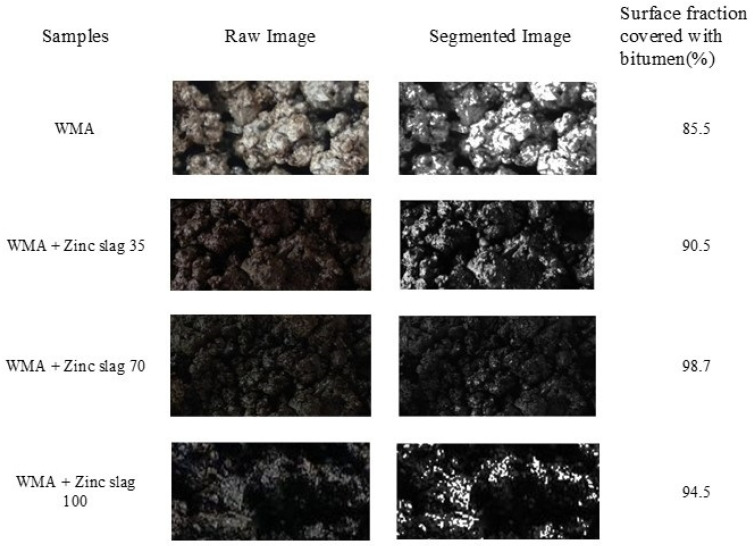
The results of the image analysis of Boiling Test samples.

As mentioned in the previous sections, digital image analysis was used to quantify the otherwise qualitative results of the boiling test, the quantity of interest being the portion of the aggregates stripped from the bituminous matrix. In the segmented images seen in Figure 6, the black represents the aggregates coated with bitumen, while the white zones show the stripped aggregates that have lost their bitumen coating. As seen in the figure, replacing a fraction of the mineral filler with slag filler reduced stripping. Among the three replacement rates, 70% zinc slag gave the most desirable performance.

**Figure 7:**
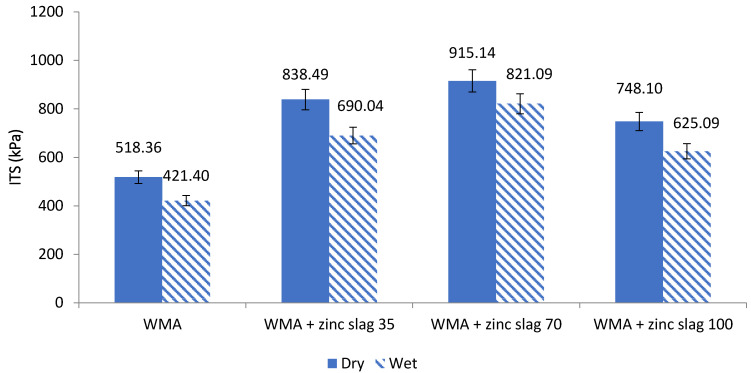
ITS diagram of the asphalt samples in dry and saturated states.

**Figure 8:**
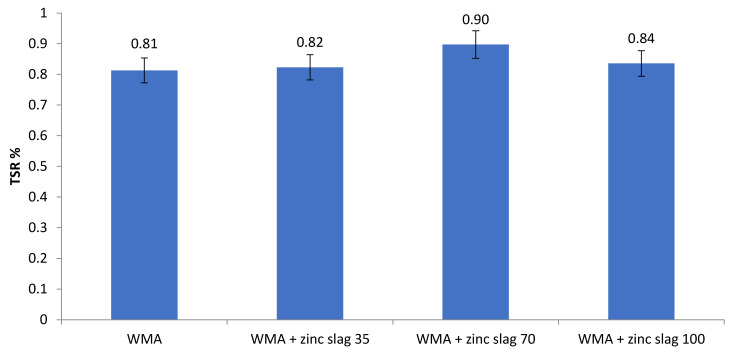
TSR values for asphalt samples.

The results of the experiment conducted according to the AASHTO T283 standard are presented in Figure 7 and Figure 8. Figure 7 shows the amount of tensile strength in the ITS test for different samples in both wet and dry conditions. As seen in the figure, replacing mineral filler with zinc slag increased the tensile strength, while the 70% replacement rate gave the most desirable results. For all mixture types, the dry strength of the specimens was higher than the saturated strength. With 70% of the mineral filler replaced by zinc slag, the indirect tensile strength increased 76% at the dry state and by 94% at the wet state.

As seen in Figure 8, the TSR values of all specimens were higher than 80%, with an increase of the TSR with zinc slag incorporation. While the unmodified WMA mixtures containing only mineral filler exceeded the 80% threshold marginally, 70% filler replacement with slag resulted in a TSR of 90%. Like the previous tests, the 70%-slag specimens performed better than all others.

**Figure 9:**
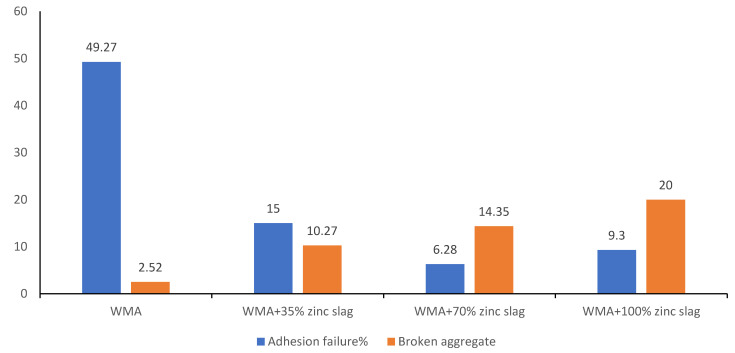
Percentage of adhesion failure and broken aggregate in samples tested by ITS.

Figure 9 shows the percentage of adhesion failure and the percentage of broken aggregates in the specimens AASHTO T283 freeze–thaw test. As seen in the figure, adhesion failure was significantly reduced by zinc slag incorporation, especially at a 70% replacement rate that showed the best results. With the aggregate–bitumen bond being strengthened by zinc slag addition, aggregate fracture was increased as expected. At a 35% replacement rate, the adhesion failure was reduced compared to the control mixture, but still, the portion of fractures occurring on the bitumen was greater than those occurring on the aggregates. At 100% filler replacement, both types of failures were higher than 70%-replacement specimens, while the ratio of aggregate fracture to adhesion failure was smaller.

**Figure 10:**
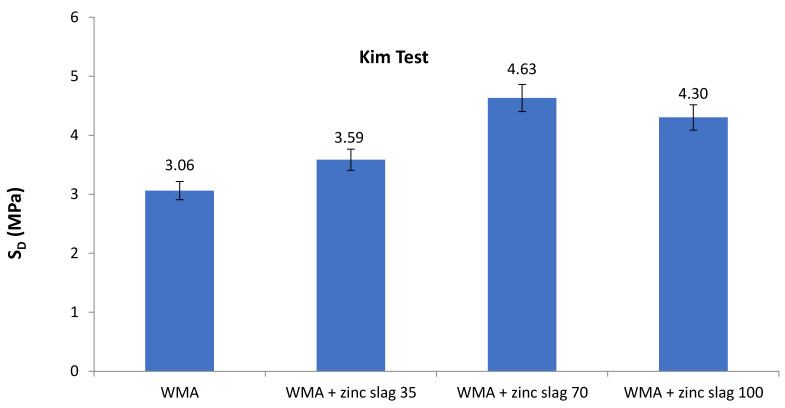
Deformation strength test values for different samples.

Figure 10 presents the results of the deformation test results that are meant to represent the rutting resistance of the WMA mixtures. The deformation strength of WMA mixtures was improved by filler replacement, as seen in the figure, and the 70%-replacement specimens performed better than other specimens. This means that the mastic film around the aggregate in the mixture containing 70% zinc slag and 30% lime filler provided the most stable WMA matrix to withstand rutting.

**Figure 11:**
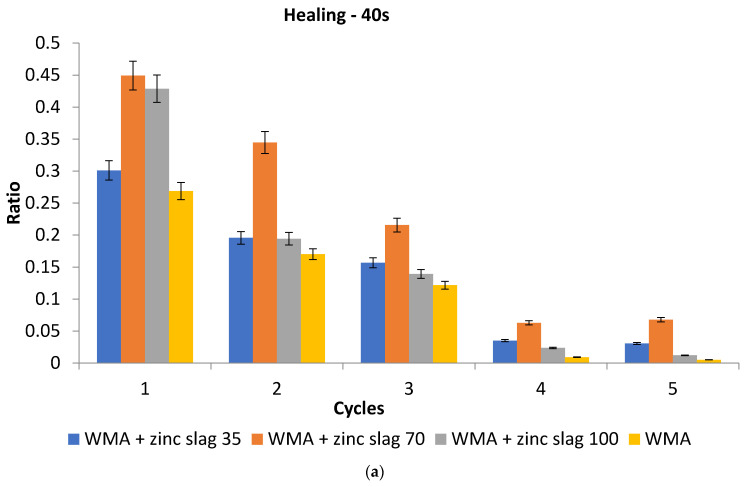

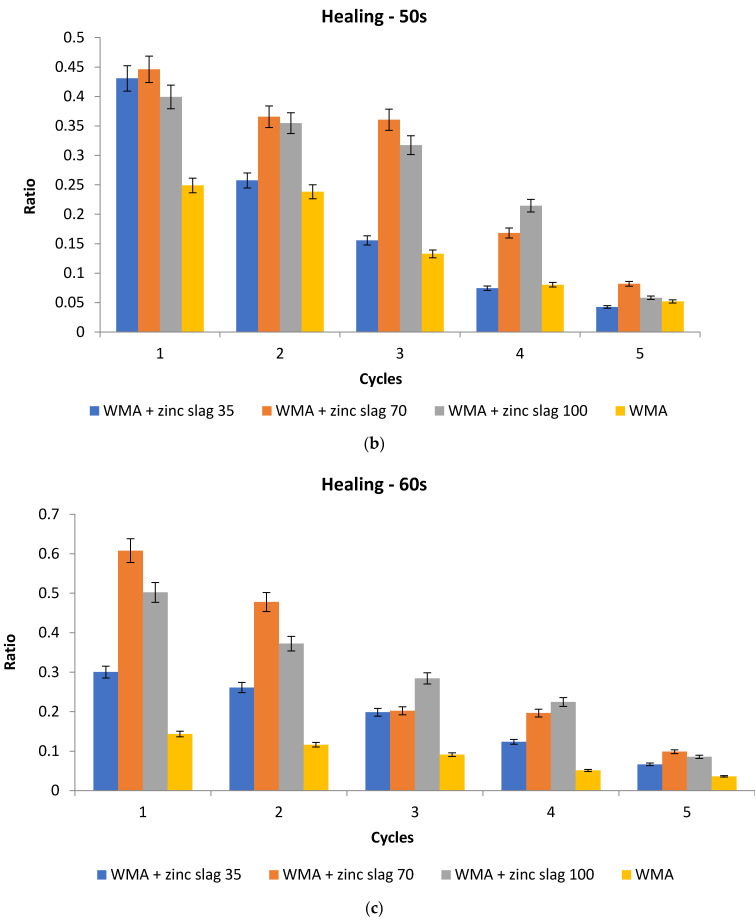
The healing of the samples under microwave heating at (**a**) 40-s, (**b**) 50-s, and (**c**) 60-s cycle durations.

Figure 11 shows healing ratios for different specimen types at two induction heating durations. As expected and shown in the figure, the strength recovery is reduced at each cycle, such that the amount of regained strength at each cycle is smaller than the preceding. With cycle durations of 40 and 60 s, the slag-containing specimens showed a higher recovery ratio than the control at any number of cycles. With 50-s cycles, only the 35%-replacement specimens showed lower strength recovery than the control at fourth and fifth cycles, while the 70% and 100%-replacement specimens still showed significantly better healing performance than the control. The 70%-replacement specimens showed the most desirable performance at all cycle numbers and durations. On average, the strength recovery ratio of asphalt mixtures containing 70% slag filler after five healing cycles were 3.8-times higher than the control specimens. Considering the results of the healing test, it seems safe to conclude that replacing 70–100% of the mineral filler with zinc slag filler can increase the self-healing capability of WMA specimens. A 60-s heating duration resulted in the highest level of healing at all slag replacement rates.

**Figure 12:**
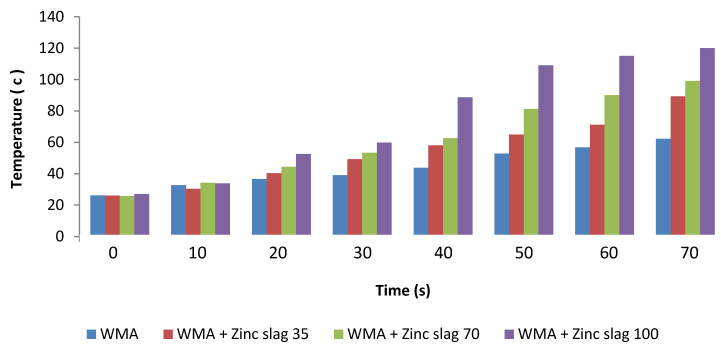
Changes in sample temperature (mean temperature) against microwave heating time.

Figure 12 shows the average temperature of the specimens at different microwave induction heating durations. As the figure reveals, increasing zinc slag content in the mixture enhanced the heat generation capability, such that the samples with 100% zinc slag filler had a 73% higher average surface temperature than the control specimens.

## 4. Discussion

As seen in the results section, replacing a fraction of mineral filler with zinc slag improved the performance of the WMA mixtures. In the case of the materials and mixtures used in this study, the texture and chemical compositions of zinc slag and mineral filler were very different. Therefore, incorporating zinc slag in the mixture required changes in mixing proportions that would result in a difference in performance. Previous studies have emphasized the critical role of filler on energy parameters in the mastic-aggregate system, essentially the asphalt mixture, thus affecting fracture and moisture-resistance behaviors [62,63]. Also, the binder–filler interaction is one of the two principal factors that govern the cohesive strength of asphalt mastic, the other being the binder characteristics [64]. Better mastic cohesion is associated with lower rutting susceptibility and potentially higher moisture resistance.

The characteristics of mastic, particularly the presence of filler, define the mixture’s moisture resistance and determine the type of failure (adhesive or cohesive) [64]. It is inferred from the results of this research that both the binder–aggregate adhesion and the mastic cohesion are improved by the incorporation of zinc slag. The adhesion failure analyses showed a reduction of adhesion failure with the incorporation of zinc slag at all replacement rates. At low zinc content (35% replacement), adhesion failure was reduced compared with the control mixtures, but it still seemed to be the dominant failure form. However, this included both the adhesion failure at the binder-aggregate interface and the mastic failure. At higher replacement rates (70–100%), aggregate fracture became the dominant form of failure, indicating a significant improvement of aggregate–binder adhesion and mastic cohesion. This can be ascribed to the physical and chemical properties of zinc slag, enabling a better bitumen–filler adhesion. As the results show, increasing the amount of zinc slag filler in the mixture increased the optimum bitumen content, which can be attributed to the higher porosity of the slag. Research has indicated [62] that specific surface area and texture dramatically influence the interfacial interaction between the binder and the filler, affecting the inter-phasal adhesion and the mixture’s rheology [14]. The zinc slag has a relatively greater surface area and higher angularity, two characteristics known for improving the binder–filler adhesion [62].

All of the experiments (except for the heat generation test) involved one observation that was a better performance of the specimens in which 70% of mineral filler was replaced with zinc slag filler. Higher heat generation capability of 100%-slag mixture is expected because of the increase of electrical and thermal conductivity with slag content. Based on these results, one may conclude that 70% is an optimum replacement rate for zinc slag with respect to Marshal stability, stripping, tensile strength, TSR, deformation strength, aggregate–binder adhesion, and healing capacity. However, this observation cannot necessarily be generalized. Let us revisit the main trend observed in the experimental results: mixture performances improved with increasing zinc slag content up to 70% of the total filler volume, while the mixtures containing only slag as filler showed slightly lower performance than those containing 70% slag. This observation implies that in addition to the changes in mixing proportions caused by filler replacement, the zinc slag, and the mineral filler may have coupled effect on the asphalt mixture’s characteristics. Considering the chemical compositions of the mineral and slag fillers (given in Table 4), replacing a large portion of mineral filler with zinc slag introduced high amounts of ZnO into the mixture, remarkably reducing the CaO and SiO_2_ contents. SiO_2_ and CaO generally increase the likelihood of binder–filler adhesion failure in the presence of moisture, especially the silica content of the filler (or aggregate) contribute to moisture damage significantly [14]. The improved moisture resistance with filler replacement is partially attributed to the chemical composition of the zinc slag. However, research has shown that in the absence of water, siliceous compounds in mineral filler could promote the cohesion of the asphalt mastic component of the mixture due to its affinity with the binder’s hydrocarbon chains [65]. An excessive amount of the low-silica zinc slag in the mixture can lead to a shift in the filler–binder adhesive bond energy in the positive direction, hence reducing the adhesion and weakening the mastic component of the mixture. The results of the tensile strength test support the possibility of such an effect, as the addition of high volumes of zinc slag led to a significantly greater increase of tensile strength at the wet state than the dry state.

The healing test results showed an overall enhancement of healing performance with slag replacement, especially at high rates of replacement and long heating durations. However, the heat generation test results indicated that the slag-containing specimens achieved considerably higher temperatures at the same heating duration. Therefore, it is hard to conclude that zinc slag incorporation improves the healing capability of the asphalt mixture, or the observed improvement of strength recovery is mainly attributed to the enhanced heat generation. The net effect of replacing mineral filler with zinc slag, partially or entirely, was improved self-healing capability under microwave induction heating, but the exact mechanism of the effect is yet to be determined. However, it should be noted that the 100%-slag specimens achieved higher temperatures than the 70%-slag specimens but exhibited lower strength recovery values. This observation implies that the zinc slag itself has a positive effect on healing capability that should be distinguished from that of the higher heat generation. The better performance of 70%-slag specimens in all other experiments strengthens the implication that the zinc slag enhances the WMA’s ability to recover, and its effect is the highest at the optimum replacement rate. Strength recovery was found to increase with heating duration, but previous studies [66] argue that the optimum heating duration varies with mixture type. One of the concerns in induction heating is the formation of heat concentration spots where the asphalt can be overheated and cause the bitumen to flow out of the specimen [66]. Heat concentration occurs due to heterogeneity of the mixture and can be exacerbated in mixtures containing metallic or electrically conductive additives if the additive is not properly dispersed [67]. No sign of excessive or abnormal heat concentration or bitumen outflow was observed in the heat generation experiments, and the heating pattern of slag-containing specimens was similar to control specimens.

Regarding the foregoing discussions and the experimental results, it is worth mentioning that the future applications of zinc slag in asphalt mixtures should consider the tradeoff between performance improvement by slag incorporation and the potential challenges associated with it, and thereby select a material combination that best fits the intended purpose. It was seen in the results that there might be an optimum zinc slag content with respect to certain performance indicators. However, it is also noteworthy that increasing the replacement rate beyond this threshold reduced the performance only relative to the—potentially—optimum content. On top of that, the optimum replacement threshold is material-specific and tends to change with variation in mixture components. Therefore, 100% replacement of mineral filler with zinc slag is an option to be considered to improve performance while maximizing the environmental and economic benefits of the practice.

## 5. Conclusions

This study investigated how the healing capability of warm mix asphalt (WMA) mixture under microwave induction heating and performance characteristics were influenced if the mineral filler is partially or entirely replaced with zinc slag. Performance was evaluated in terms of indicators representing rutting susceptibility, and moisture resistance. To this end, appropriate specimens made with 0, 35, 70, and 100% of the mineral filler replaced by zinc slag were prepared and tested according to the standards.

- Zinc-containing mixtures possessed higher Marshall stability and flow values than the control samples.
- The results of stripping and moisture resistance tests indicated that the presence of zinc slag in the WMA mixtures improved aggregate–binder adhesion as well as the cohesive strength of the mixture’s mastic component.
- Replacing the mineral filler with zinc slag at all rates was found to increase moisture resistance and reduce the rutting susceptibility of the WMA mixtures.
- The slag-containing specimens showed significantly higher heat generation capability under microwave induction without noticeable heat concentration or non-uniformity.
- The overall self-healing capacity of the mixtures was improved by zinc slag, the strength recovery of slag-containing specimens being considerably higher than the control.
- While the strength recovery improvement by slag incorporation is partially attributed to higher heat generation ability, zinc slag was found to positively affect WMA’s healing capacity under induction heating independent of the temperature.
- Strength recovery increased with the heating duration within the studied range of 40–60 s.
- Mixtures with 70% filler replacement displayed the highest Marshall stability and flow, ITS, TSR, and rutting resistance. Furthermore, replacing 70% of the mineral filler with zinc slag resulted in better aggregate–bitumen adhesion and self-healing capability compared to the other replacement rates. Increasing the filler replacement rate led to increased heating capability under microwave induction; however, the strength recovery ratio in the healing test was the highest at a 70% replacement rate.
- The results of this study revealed that the rate at which mineral filler is replaced with zinc slag could have an optimum value, beyond which the performance does not experience further improvement and can relatively degrade. Given the materials and method used in this research, 70% can be considered as the optimum rate of replacing mineral filler with zinc slag. However, care must be taken in generalizing or adopting this finding because this optimum value is specific to materials and mix design.
- Investigating the influence of zinc slag on energy parameters of the asphalt mixture components by future studies can create insights into the mechanisms involved in the interactions between the components of these mixtures, especially in the presence of water. Also, temperature-controlled self-healing tests can distinguish between the healing improvement caused by the mixture’s increased heat generation capability or enhanced healing capacity.

## Figures and Tables

**Table 1:** Bitumen Properties.

Bitumen Properties	Bitumen (60/70)	Test Method	Unit
Specific gravity	1.017	ASTM D70	g/cm^3^
Penetration grade at 25 °C	65	ASTM D5	0.1 mm
Softening point	49.8	ASTM D36	°C
Ductility at 25 °C	>100	ASTM D113	cm
Flash point	334	ASTM D92	°C

**Table 2:** Marshall mix design for the WMA mixtures used in this research.

	WMA	WMA + Zinc Slag 35	WMA + Zinc Slag 70	WMA + Zinc Slag 100
Mixture type *	100/0	65/35	30/70	0/100
Optimum Bitumen Content (%)	5.2%	5.3%	5.4%	5.5%
Marshall stability (KN)	9.6	14.25	14.7	14.4
Marshal flow (mm)	3.2	3.4	3.5	3.6
Bulk specific gravity	2.33	2.32	2.31	2.29
VMA ^1^	14.9	15	15.1	15.2
VFA ^2^	73.1	73.3	73.5	73.7

* Asphalt content (Lime filler %/Zinc Slag filler %). ^1^ void of mineral aggregates. ^2^ void filled with asphalt binder.

**Table 3:** The Properties of the Aggregate.

Description	Test Results	Test Method	Unit
LA abrasion value	22.3	AASHTO T96	-
Particle shape Flakiness index	16	BS 812	-
The Percentage of Fractured Particles in Coarse Aggregate	93	ASTM D5821	-
Maximum water absorption of coarse aggregate	2.2	AASHTO T85	-
Maximum water absorption of fine aggregate	2.4	AASHTO T84	-
SSD specific gravity of coarse aggregate	2.59	ASTM C127	g/cm^3^
SSD specific gravity of fine aggregate	2.32	ASTM C128	g/cm^3^

**Table 4:** Chemical composition (XRF).

Composition	CaO	SiO_2_	Fe_2_O_3_	Al_2_O_3_	MgO	SO_3_	K_2_O	ZnO	Other
Filler	Content (% wt.)								
Mineral	49.05	10.19	0.71	1.20	0.77	0.10	0.40	-	37.47
Slag	0.22	0.73	1.88	-	-	-	1.20	60.05	35.92

**Table 5:** Marshall mix design results.

	WMA	WMA + Zinc Slag 35	WMA + Zinc Slag 70	WMA + Zinc Slag 100
Mixture type *	100/0	65/35	30/70	0/100
O.B.C %	5.2%	5.3%	5.4%	5.5%
Marshall stability (KN)	9.6	14.25	14.7	14.4
Marshal flow (mm)	3.2	3.4	3.5	3.6
Bulk specific gravity	2.33	2.32	2.31	2.29

* Asphalt content (Lime filler %/Zinc Slag filler %).

## Data Availability

Some or all data, models, or code that support the findings of this study can be provided by the corresponding author upon request.

## References

[1] Thøgersen F., Colette G., Josef S., Pierre H., Yannick D., Cyrille C., Anita B., Broere P., Bizjak K.F., Hellman F. (2013). Recycling of road materials into new unbound road layers–main practice in selected European countries. Road Mater. Pavement Des..

[2] Arabani M., Mirabdolazimi S.M., Sasani A.R. (2010). The effect of waste tire thread mesh on the dynamic behaviour of asphalt mixtures. Constr. Build. Mater..

[3] Yalcin E. (2021). Effects of microwave and induction heating on the mechanical and self-healing characteristics of the asphalt mixtures containing waste metal. Constr. Build. Mater..

[4] González A., Norambuena-Contreras J., Storey L., Schlangen E. (2018). Self-healing properties of recycled asphalt mixtures containing metal waste: An approach through microwave radiation heating. J. Environ. Manag..

[5] Do H.S., Mun P.H., Keun R.S. (2008). A study on engineering characteristics of asphalt concrete using filler with recycled waste lime. Waste Manag..

[6] Ajam H., Gómez-Meijide B., Artamendi I., Garcia A. (2018). Mechanical and healing properties of asphalt mixes reinforced with different types of waste and commercial metal particles. J. Clean. Prod..

[7] Xue Y., Hou H., Zhu S., Zha J. (2009). Utilization of municipal solid waste incineration ash in stone mastic asphalt mixture: Pavement performance and environmental impact. Constr. Build. Mater..

[8] Javed S., Lovell C.W., Wood L.E. (1994). Waste foundry sand in asphalt concrete. Transp. Res. Rec..

[9] Franesqui M.A., Yepes J., García-González C. (2017). Top-down cracking self-healing of asphalt pavements with steel filler from industrial waste applying microwaves. Constr. Build. Mater..

[10] Norambuena-Contreras J., Gonzalez A., Concha J.L., Gonzalez-Torre I., Schlangen E. (2018). Effect of metallic waste addition on the electrical, thermophysical and microwave crack-healing properties of asphalt mixtures. Constr. Build. Mater..

[11] González A., Norambuena-Contreras J., Storey L., Schlangen E. (2018). Effect of RAP and fibers addition on asphalt mixtures with self-healing properties gained by microwave radiation heating. Constr. Build. Mater..

[12] da Silva W.R., da Silva F.B.V., Araújo P.R.M., Nascimento C.W.A.D. (2017). Assessing human health risks and strategies for phytoremediation in soils contaminated with As, Cd, Pb, and Zn by slag disposal. Ecotoxicol. Environ. Saf..

[13] Qing Z., Qi-Cheng L., Peng L., Chuan-Sheng C., Jiang-Rong K. (2020). Study on modification mechanism of nano-ZnO/polymerised styrene butadiene composite-modified asphalt using density functional theory. Road Mater. Pavement Des..

[14] Taherkhani H., Kamsari S.V. (2020). Evaluating the properties of zinc production wastes as filler and their effects on asphalt mastic. Constr. Build. Mater..

[15] Barri K., Jahangiri B., Davami O., Buttlar W.G., Alavi A.H. (2020). Smartphone-based molecular sensing for advanced characterization of asphalt concrete materials. Measurement.

[16] Ouyang C., Wang S., Zhang Y., Zhang Y. (2006). Improving the aging resistance of asphalt by addition of Zinc dialkyldithiophosphate. Fuel.

[17] Hamedi G.H., Nejad F.M., Oveisi K. (2016). Estimating the moisture damage of asphalt mixture modified with nano zinc oxide. Mater. Struct..

[18] Saltan M., Terzi S., Karahancer S. (2019). Mechanical Behavior of Bitumen and Hot-Mix Asphalt Modified with Zinc Oxide Nanoparticle. J. Mater. Civ. Eng..

[19] Aktaş B., Şevket A. Laboratory Evaluation on Waste Slag Produced Zinc Industry as Mineral Filler in Stone Mastic Asphalt. Proceedings of the 6th Eurasphalt & Eurobitume Congress.

[20] Larsen O.R., Moen O., Robertus C., Koenders B.G. WAM Foam asphalt production at lower operating temperatures as an environmentally friendly alternative to HMA. Proceedings of the 3rd EUasphalt and Eurobitumen congress.

[21] Fakhri M., Ahmadi A. (2017). Recycling of RAP and steel slag aggregates into the warm mix asphalt: A performance evaluation. Constr. Build. Mater..

[22] Liu Q., Yu W., Wu S., Schlangen E., Pan P. (2017). A comparative study of the induction healing behaviors of hot and warm mix asphalt. Constr. Build. Mater..

[23] Cucalon L.G., Yin F., Martin A.E., Arambula E., Estakhri C., Park E.S. (2016). Evaluation of Moisture Susceptibility Minimization Strategies for Warm-Mix Asphalt: Case Study. J. Mater. Civ. Eng..

[24] Ziari H., Moniri A., Imaninasab R., Nakhaei M. (2019). Effect of copper slag on performance of warm mix asphalt. Int. J. Pavement Eng..

[25] Bennert T. (2021). Performance Evaluation of Asphalt Mixtures Statewide.

[26] Advanced Asphalt Technologies, LLC (2011). A Manual for Design of Hot Mix Asphalt With Commentary.

[27] Javilla B., Fang H., Mo L., Shu B., Wu S. (2017). Test evaluation of rutting performance indicators of asphalt mixtures. Constr. Build. Mater..

[28] Polaczyk P., Ma Y., Xiao R., Hu W., Jiang X., Huang B. (2021). Characterization of aggregate interlocking in hot mix asphalt by mechanistic performance tests. Road Mater. Pavement Des..

[29] Abdelsalam M., Yue Y., Khater A., Luo D., Musanyufu J., Qin X. (2020). Laboratory Study on the Performance of Asphalt Mixes Modified with a Novel Composite of Diatomite Powder and Lignin Fiber. Appl. Sci..

[30] Xu S., Xiao F., Amirkhanian S., Singh D. (2017). Moisture characteristics of mixtures with warm mix asphalt technologies—A review. Constr. Build. Mater..

[31] Fakhri M., Hosseini S.A. (2017). Laboratory evaluation of rutting and moisture damage resistance of glass fiber modified warm mix asphalt incorporating high RAP proportion. Constr. Build. Mater..

[32] Yu H., Chen Y., Wu Q., Zhang L., Zhang Z., Zhang J., Miljković M., Oeser M. (2020). Decision support for selecting optimal method of recycling waste tire rubber into wax-based warm mix asphalt based on fuzzy comprehensive evaluation. J. Clean. Prod..

[33] Zhu J., Zhang K., Liu K., Shi X. (2019). Performance of hot and warm mix asphalt mixtures enhanced by nano-sized graphene oxide. Constr. Build. Mater..

[34] Kumar P., Chandra S., Bose S. (2004). Rheology of the Polymer Modified Binders. Highw. Res. Bull..

[35] Kringos N., Schmets A., Pauli T., Scarpas T. A finite element base chemomechanical model to stimulate healing in bitumen. Proceedings of the International Workshop on Chemo-mechanics of Bituminous materials.

[36] García Á. (2012). Self-healing of open cracks in asphalt mastic. Fuel.

[37] García A., Bueno M., Norambuena-Contreras J., Manfred N.P. (2013). Induction healing of dense asphalt concrete. Constr. Build. Mater..

[38] Lesueur D. (2009). The colloidal structure of bitumen: Consequences on the rheology and on the mechanisms of bitumen modification. Adv. Colloid Interface Sci..

[39] Bhasin A., Palvadi S., Little D.N. (2011). Influence of Aging and Temperature on Intrinsic Healing of Asphalt Binders. Transp. Res. Rec..

[40] Gómez-Meijide B., Ajam H., González P.L., Garcia A. (2016). Effect of air voids content on asphalt self-healing via induction and infrared heating. Constr. Build. Mater..

[41] Menozzi A., Garcia A., Partl M.N., Tebaldi G., Schuetz P. (2015). Induction healing of fatigue damage in asphalt test samples. Constr. Build. Mater..

[42] Liu Q., Wu S., Schlangen E. (2013). Induction heating of asphalt mastic for crack control. Constr. Build. Mater..

[43] Gao J. (2014). Carbon Fiber-Cement Emulsified Asphalt Mortar with Tropical Design and Microwave Deicing Function Research. Master’s Thesis.

[44] Sassani A., Arabzadeh A., Ceylan H., Kim S., Gopalakrishnan K., Taylor P.C., Nahvi A. (2019). Polyurethane-carbon microfiber composite coating for electrical heating of concrete pavement surfaces. Heliyon.

[45] Galindo B., Benedito A., Ramos F., Gimenez E. (2016). Microwave heating of polymers: Influence of carbon nanotubes dispersion on the microwave susceptor effectiveness. Polym. Eng. Sci..

[46] Arabzadeh A., Notani M.A., Zadeh A.K., Nahvi A., Sassani A., Ceylan H. (2019). Electrically conductive asphalt concrete: An alternative for automating the winter maintenance operations of transportation infrastructure. Compos. Part B Eng..

[47] Arabzadeh A., Ceylan H., Kim S., Gopalakrishnan K., Sassani A. (2016). Superhydrophobic Coatings on Asphalt Concrete Surfaces: Toward Smart Solutions for Winter Pavement Maintenance. Transp. Res. Rec..

[48] Wuori A.F. (1993). Ice-Pavement Bond Disbonding: Surface Modification and Disbonding.

[49] Osborne T.L., Hutcheson W.R. (1989). Asphaltic Compositions and Uses Therefor. U.S. Patent.

[50] Nabiun N., Khabiri M.M. (2016). Mechanical and moisture susceptibility properties of HMA containing ferrite for their use in magnetic asphalt. Constr. Build. Mater..

[51] Abo-Qudais S. (2007). The effects of damage evaluation techniques on the prediction of environmental damage in asphalt mixtures. Build. Environ..

[52] Mogawer W.S., Alexander A.J., Hussain B.U. (2011). Evaluating the effect of warm-mix asphalt technologies on moisture characteristics of asphalt binders and mixtures. Transp. Res. Rec..

[53] Lottman R.P. (1982). Predicting Moisture-Induced Damage to Asphaltic Concrete Field Evaluation.

[54] Fakhri M., Javadi S., Sedghi R., Arzjani D., Zarrinpour Y. (2019). Effects of deicing agents on moisture susceptibility of the WMA containing recycled crumb rubber. Constr. Build. Mater..

[55] Kim K.W., Doh Y.S., Amrikhanian S.N. (2004). Feasibility of deformation strength for estimation of rut resistance of asphalt concrete. Road Mater. Pavement Des..

[56] Doh Y.S., Yun K.K., Amirkhanian S.N., Kim K.W. (2007). Framework for developing a static strength test for measuring deformation resistance of asphalt concrete mixtures. Constr. Build. Mater..

[57] Fakhri M., Maleki H., Hosseini S.A. (2017). Investigation of different test methods to quantify rutting resistance and moisture damage of GFM-WMA mixtures. Constr. Build. Mater..

[58] Norambuena-Contreras J., Gonzalez-Torre I. (2017). Influence of the microwave heating time on the self-healing properties of asphalt mixtures. Appl. Sci..

[59] Zhao H., Zhong S., Zhu X., Chen H. (2017). High-efficiency heating characteristics of ferrite-filled asphalt-based composites under microwave irradiation. J. Mater. Civ. Eng..

[60] Fakhri M., Javadi S., Sedghi R., Sassani A., Arabzadeh A., Bahmai B.B. (2021). Microwave Induction Heating of Polymer-Modified Asphalt Materials for Self-Healing and Deicing. Sustainability.

[61] Xu S., García A., Su J., Liu Q., Tabaković A., Schlangen E. (2018). Self-Healing Asphalt Review: From Idea to Practice. Adv. Mater. Interfaces.

[62] Tan Y., Guo M. (2013). Using surface free energy method to study the cohesion and adhesion of asphalt mastic. Constr. Build. Mater..

[63] Lytton R.L., Masad E.A., Zollinger C., Bulut R., Little D.N. (2005). Measurements of Surface Energy and Its Relationship to Moisture Damage.

[64] Kim Y.-R., Little D.N., Lytton R.L. (2003). Fatigue and Healing Characterization of Asphalt Mixtures. J. Mater. Civ. Eng..

[65] Arabani M., Hamedi G.H. (2014). Using the surface free energy method to evaluate the effects of liquid antistrip additives on moisture sensitivity in hot mix asphalt. Int. J. Pavement Eng..

[66] Xu S., Liu X., Tabaković A., Schlangen E. (2021). The Prospect of Microwave Heating: Towards a Faster and Deeper Crack Healing in Asphalt Pavement. Processes.

[67] Sassani A., Arabzadeh A., Ceylan H., Kim S., Sadati S.S.M., Gopalakrishnan K., Taylor P.C., Abdualla H. (2018). Carbon fiber-based electrically conductive concrete for salt-free deicing of pavements. J. Clean. Prod..

